# Somatic mutation effects diffused over microRNA dysregulation

**DOI:** 10.1093/bioinformatics/btad520

**Published:** 2023-08-25

**Authors:** Hui Yu, Limin Jiang, Chung-I Li, Scott Ness, Sara G M Piccirillo, Yan Guo

**Affiliations:** Department of Public Health, Sylvester Comprehensive Cancer Center, University of Miami, Miami, FL 33136, U.S.A; Department of Public Health, Sylvester Comprehensive Cancer Center, University of Miami, Miami, FL 33136, U.S.A; Department of Statistics, National Cheng Kung University, Tainan 701401, Taiwan; Comprehensive Cancer Center, University of New Mexico, Albuquerque, NM 87109, United States; Comprehensive Cancer Center, University of New Mexico, Albuquerque, NM 87109, United States; Department of Public Health, Sylvester Comprehensive Cancer Center, University of Miami, Miami, FL 33136, U.S.A

## Abstract

**Motivation:**

As an important player in transcriptome regulation, microRNAs may effectively diffuse somatic mutation impacts to broad cellular processes and ultimately manifest disease and dictate prognosis. Previous studies that tried to correlate mutation with gene expression dysregulation neglected to adjust for the disparate multitudes of false positives associated with unequal sample sizes and uneven class balancing scenarios.

**Results:**

To properly address this issue, we developed a statistical framework to rigorously assess the extent of mutation impact on microRNAs in relation to a permutation-based null distribution of a matching sample structure. Carrying out the framework in a pan-cancer study, we ascertained 9008 protein-coding genes with statistically significant mutation impacts on miRNAs. Of these, the collective miRNA expression for 83 genes showed significant prognostic power in nine cancer types. For example, in lower-grade glioma, 10 genes’ mutations broadly impacted miRNAs, all of which showed prognostic value with the corresponding miRNA expression. Our framework was further validated with functional analysis and augmented with rich features including the ability to analyze miRNA isoforms; aggregative prognostic analysis; advanced annotations such as mutation type, regulator alteration, somatic motif, and disease association; and instructive visualization such as mutation OncoPrint, Ideogram, and interactive mRNA–miRNA network.

**Availability and implementation:**

The data underlying this article are available in MutMix, at http://innovebioinfo.com/Database/TmiEx/MutMix.php.

## 1 Introduction

The advances in high-throughput sequencing have fueled explosive research progress in cancer genomics, yielding enormous variant/mutation data for up to 10 000 cancer patients in a typical consortium project. In such a context, there is a pressing need to elucidate gene mutations’ functional impacts, ideally relating them to pathophysiology or prognosis (Samuels *et al.* 2022). Mutations are a well-known causal factor of cancers with a wide range of cascading detrimental effects. Based on the Central Dogma of molecular biology, mutations should cast their impacts on the transcriptomes to affect proteins, the key player underlying biological processes, and pathways. For this reason, somatic motifs, which are binding sequences suppressed or created by somatic mutations, are identified and characterized to facilitate cancer research (Jiang *et al.* 2022). Due to data rarity at the individual mutation level, researchers often aggregate mutations into meaningful units and infer the transcriptomic impact for a mutation type, cluster, or gene (Gamazon *et al.* 2015, Jia and Zhao 2017).

Typically, mutations may dysregulate gene expression levels, often affecting a large number of genes (Jia and Zhao 2017, Ping *et al.* 2020). In previous studies (Jia and Zhao 2017, Ping *et al.* 2020) that tried to associate mutation with gene expression dysregulation, the common approach was to assign the samples into mutant and wild-type classes based on a gene’s mutation status and then conduct differential expression (DE) analyses between the different mutation classes. Such an approach is effective yet prone to potential statistic biases due to sample size and class imbalance. That is, when dichotomizing any dataset of abundant genes, DE analyses may yield statistically significant yet biologically irrelevant results. To address such a pitfall, we herein developed a permutation-based statistical framework and applied it to associate cancer mutations and microRNAs (miRNAs) expression changes.

In contrast to mRNAs that code for proteins, miRNAs are a species of small, noncoding RNAs. MiRNAs have been accredited with critical regulatory importance (Tu *et al.* 2009) and implicated with disease phenotypes (Jiang *et al.* 2009, Huang *et al.* 2019), especially tumorigenesis (Bhattacharya and Cui 2016). While miRNAs’ regulation mechanisms and disease associations have been extensively investigated, no research efforts have been directed at their dysregulations due to mutations. We hypothesized that miRNAs may effectively diffuse somatic mutation impacts to broad cellular processes and ultimately manifest disease and dictate the prognosis in cancer patients. Henceforth, our permutation-based statistical framework was applied in a pan-cancer miRNA expression dataset to infer mRNA–miRNA associations. Here, an mRNA–miRNA association refers to a significant expression change of an miRNA in response to the mutations in an mRNA. The identified mRNA–miRNA association data were further examined against a multitude of functional evidence, including somatic motifs (Jiang *et al.* 2022), experimentally validated mRNA–miRNA regulations (Tong *et al.* 2019), and prognosis relevance in terms of aggregative expression score (Ye *et al.* 2020). In Section 3, we will present the major findings from the pan-cancer study and thoroughly demonstrate the effectiveness of our framework.

## 2 Materials and methods

### 2.1 Precomputation of mRNA–miRNA associations followed by prognosis analyses

We obtained somatic mutations from the Multi-Center Mutation Calling in Multiple Cancers (MC3) project of The Cancer Genome Atlas (TCGA) (Ellrott *et al.* 2018). Additionally, miRNA sequencing data and patient survival data (TCGA-Clinical Data Resource) were obtained for the same patients as in the mutation datasets. All these data were downloaded from Genomic Data Commons (https://gdc.cancer.gov).

**Figure 1.**
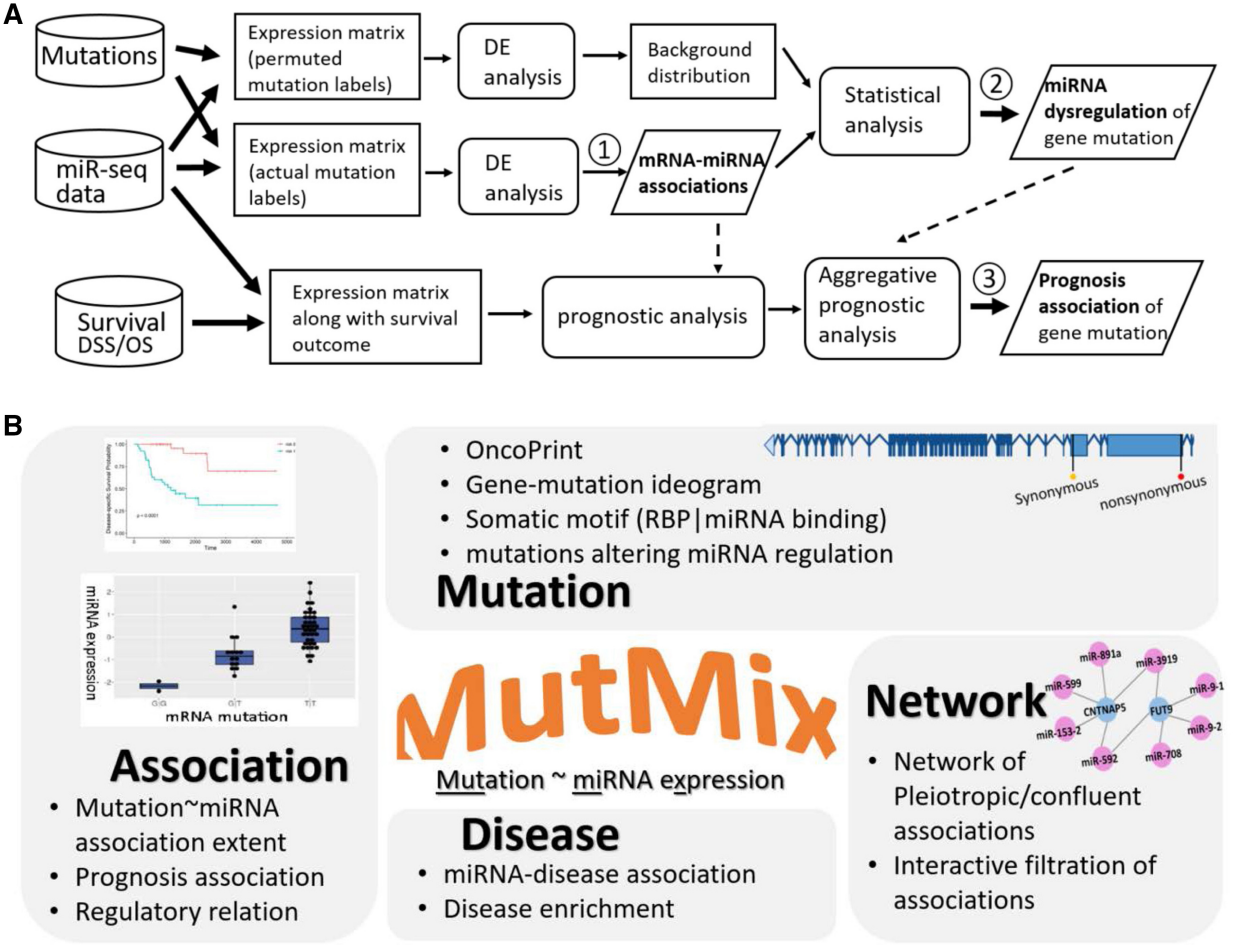
MutMix workflow and schema. (A) Workflow to elucidate the diffusion of mRNA somatic mutation effects over miRNAs and further assess the prognosis by dysregulated miRNA expression. The workflow was applied in each mutation context that was defined with respect to one cancer type and one mutated gene. The workflow consisted of three major steps, whose immediate outputs were highlighted in bold texts. At step ①, miRNA sequencing data and genomic mutation information were integrated, and a generalized linear model was employed through edgeR to discern statistically significant differential expression (DE) miRNAs. At step ②, a multitude of dysregulated miRNAs were not directly considered as a sign of great mutation impact; instead, a class-ratio-controlled permutation procedure using the same miRNA expression data yet permutated mutation labels allowed for statistical tests under three alternative null assumptions: empirical, Gaussian, and Negative Binomial. At step ③, patients’ survival data were integrated with miRNA expression through Combined Gene Expression Score (CGES), and a hypothesis test of prognosis relevance was conducted against three alternative null distributions: empirical, Gaussian, and Beta. (B) Core data of mRNA–miRNA associations and relevant annotations are organized into four sectors in the online portal MutMix: Mutation, Association, Network, and Disease.

From the raw MC3 mutation data, we leveraged ANNOVAR (Wang *et al*. 2010) to annotate the protein-coding impacts of each mutation, identifying if its location relative to the exon/intron framework and assessing if it changed the protein sequence. Mutations causing nonsynonymous changes to protein-coding sequences were considered “nonsilent.” Locus-wise mutations were aggregated to the gene level, meaning that a gene was regarded as mutated in a patient as long as at least one nonsilent mutation located within the gene body was reported for the patient. All patient samples of a cancer type were separated into two groups based on the mutation state of a particular gene (mutant versus wild-type); such a gene-specific division of patient samples was termed a “mutation context.” Hence, for each mutation context, we managed to obtain a statistic of “mutation frequency,” which equated to the percentage of mutated patients with regard to a particular gene in a particular cancer-type-specific cohort. To avoid extreme imbalance of the binary classes, we reserved only mutation contexts where the mutation frequency and nonmutation frequency were both above 5%. As a result, we compiled 703 957 cancer-by-gene mutation contexts. For each mutation context, we identified DE miRNAs (or so-called dysregulated or responsive miRNAs) between the mutated samples and the nonmutated samples (Fig. 1A, Step 1). The DE analysis was performed on an miRNA expression matrix using R package edgeR (Robinson *et al.* 2010). At a false discovery rate (estimated with the Benjamin–Hochberg method) threshold of 0.05, 20 804 mutation contexts reported one or more dysregulated miRNAs. The number of dysregulated miRNAs ranged over 40–550.

Intuitively, the quantity of dysregulated miRNAs may reflect the extent of a gene’s mutation impact. However, the quantities of dysregulated miRNAs may not be directly compared across genes or cancer types, because all mutation contexts had unequal mutation frequencies, resulting in uneven class imbalance severities. Therefore, additional statistical analyses were designed to assess a mutation context’s impact extent with the accommodation of variant mutation frequencies (Fig. 1A, Step 2). To this end, we randomly permutated sample mutation labels 1000 times for each discrete mutation frequency category and assessed the statistical significance of the total number of dysregulated miRNAs relative to the permutation-based, mutation-frequency-matched null distribution. For each cancer cohort, we separately treated 10 discrete mutation frequency categories, namely 0.05, 0.1, 0.15, 0.2, 0.25, 0.3, 0.35, 0.4, 0.45, and 0.5.

Given an observed number of dysregulated miRNAs for a particular mutation context, we identified the corresponding null distribution by rounding the actual mutation frequency up to the nearest discrete mutation frequency category; such a mapping of proper null backgrounds ensured that both sample size and class imbalance were controlled for in the statistical tests. Based on the traditional permutation test procedure, an empirical *P*-value was derived as the proportion of cases returning equivalent or more dysregulated miRNAs out of the total 1000 permutation cases. Many mutation contexts ended up with the same minimal *P*-value of .001 in this way. To discriminate the impact extent more precisely, we calculated *z*-score statistics in companion with the empirical *P*-value; the *z*-score intuitively approximated the deviation of the observed DE miRNA quantity from the average value under Gaussian null distribution. In addition, we modified the permutation-based *P*-value assessment by fitting the null distribution with the Negative Binomial model. Because of its much higher precision than the traditional permutation *P*-value, the modified *P*-value was adopted for all statistical tests in the present report.

Prognosis is a crucial element in cancer patients’ medical care. For each DE miRNA (responsive miRNA) in a mutation context, we calculated a prognostic *P*-value by fitting patients’ survival outcomes with the miRNAs expression values through the Cox proportional hazards model. Two distinct measures of survival were analyzed separately: overall survival (OS) and disease-specific survival (DSS) (Liu *et al.* 2018). Furthermore, at the gene level, we evaluated the collective prognosis of the miRNA set through the Composite Gene Expression Score (CGES) (Ye *et al.* 2020). In addition to the conventional Cox-model *P*-value, the CGES algorithm has a built-in permutation procedure to infer an unbiased *P*-value. Because the test statistics of CGES are between 0 and 1, we approximated the permutation-led CGES scores with Beta distribution and thus derived a beta-distribution *P*-value as an alternative to the CGES permutation *P*-value (Fig. 1A, Step 3).

### 2.2 Multidimension organization of mutation-associated miRNA dysregulation data in MutMix

The framework expounded above was carried out in multiomics, pan-cancer datasets from TCGA, which involved 27 tissue sites and 33 cancer types (full names and abbreviations are given in Supplementary Text S1). The pan-cancer study is organized into a web portal named MutMix. The core program of MutMix is developed in Python, and the web interface is designed in PHP and Javascript. MutMix provides multidimension perspectives to dissect enormous pan-cancer association relations between mRNA mutation and miRNA dysregulation, elucidating the diffusion of cancer mutations’ functional impacts over miRNA dysregulation networks. Revolving the central data of mRNA–mutation associations, we organize the contents of MutMix into four dimensions or sectors: Association, Mutation, Network, and Disease (Fig. 1B).

The Association sector stores data on two levels. First, responsive miRNAs along with DE statistics are collated for each mutation context, which corresponds to one mutated mRNA gene in a cancer type. Second, the multitude of dysregulated miRNAs per mutation context is converted to mutation impact extent statistics of the defining gene in the specific cancer type. An mRNA (protein-coding gene) is identified as a transcription regulator if it appears in recent compendiums of human transcription factors (Ng *et al.* 2021) or RNA-binding proteins (Hentze *et al.* 2018). MiRNA evolution conservation is distinguished into four cardinal categories according to TargetScan (McGeary *et al.* 2019). Whenever possible, the regulatory effect of a transcription factor on an miRNA is specified as activation, repression, or regulation per TransmiR (Tong *et al.* 2019). Proteins may influence miRNA expression indirectly through signaling network via affecting kinase, phosphatase, or GTPase expression. From Reactome (Gillespie *et al.* 2022) and KEGG (Kanehisa and Goto 2000), we retrieved 674 cellular pathways that each involved miRNAs as well as mRNAs, and marked them up in our result set. Prognosis characterizations include numeric statistics of the *P*-value and the hazard ratio, as well as a Kaplan–Meier curve plot that is dynamically rendered for each prognostic mutation context. A recently devised intersection visualization termed UpSet plot (Conway *et al.* 2017) is implemented to facilitate cross-cancer or cross-gene comparisons of mRNA–miRNA associations.

The Mutation sector tabulates and visualizes somatic mutations in the 33 cancer cohorts. One mutation is defined by the following minimum attributes: chromosome, chromosome position (coordinate), reference allele, and alternate allele. Both single-nucleotide variants and small indels are included. Additionally, the gene that embodies the mutation, the class of the mutation in terms of coding disruption, and the cancer cohort that presents the mutation are all indicated. Furthermore, we marked out the mutations curated in two external cancer annotation databases: SomamiR (Bhattacharya and Cui 2016) and SMDB (Jiang *et al.* 2020). SomamiR isolates and annotates cancer somatic mutations in miRNAs and their target sites that potentially alter the interactions between miRNAs and mRNAs. SMDB stores somatic motifs pertinent to transcription factors, RNA-binding proteins, and miRNAs, where a somatic motif designates an altered binding motif resulting from somatic mutations. Leveraging R package genemodel (https://cran.r-project.org/web/packages/genemodel/index.html, v1.1.0), we manifest a gene-mutation Ideogram, which illustrates the dispersal of mutations in an exon–intron ideogram of the gene body. From another angle, we implement a so-called OncoPrint (Cerami *et al.* 2012) to compare the mutation dispersal profiles of multiple genes within a same patient cohort.

The Network sector represents mRNA–miRNA associations in each cancer type in a graph (vertex-edge) schema. Because of the pleiotropic and confluent nature of massive mRNA–miRNA associations, edges going out from different mRNAs are connected into a network via common miRNA targets. The network is responsive to settings that can be tweaked by the user interactively.

Lastly, the disease sector prioritizes the most relevant human diseases that are enriched in the mutation-dysregulated miRNAs. Diseases associated with the dysregulated miRNAs are ascertained by the Human microRNA Disease Database (HMDD v3.0; Huang *et al.* 2019). The disease enrichment analysis is powered by R package clusterProfiler v4.0 (Wu *et al.* 2021).

### 2.3 Novel webserver MutMix

The immense mRNA–miRNA associations as well as the opulent annotation data generated for all cancer types from the pan-cancer study are organized into a web portal named MutMix, which can be accessed freely at http://innovebioinfo.com/Database/TmiEx/MutMix.php. The whole work was implemented in Linux Shell, R, and PHP scripts. The Perl application ANNOVAR (Wang *et al.* 2010) was employed to annotate deleterious scores for nonsynonymous single-nucleotide variants in TCGA data.

## 3 Results

### 3.1 Accommodating class imbalance and sample size in the assessment of miRNA dysregulation

**Figure 2.**
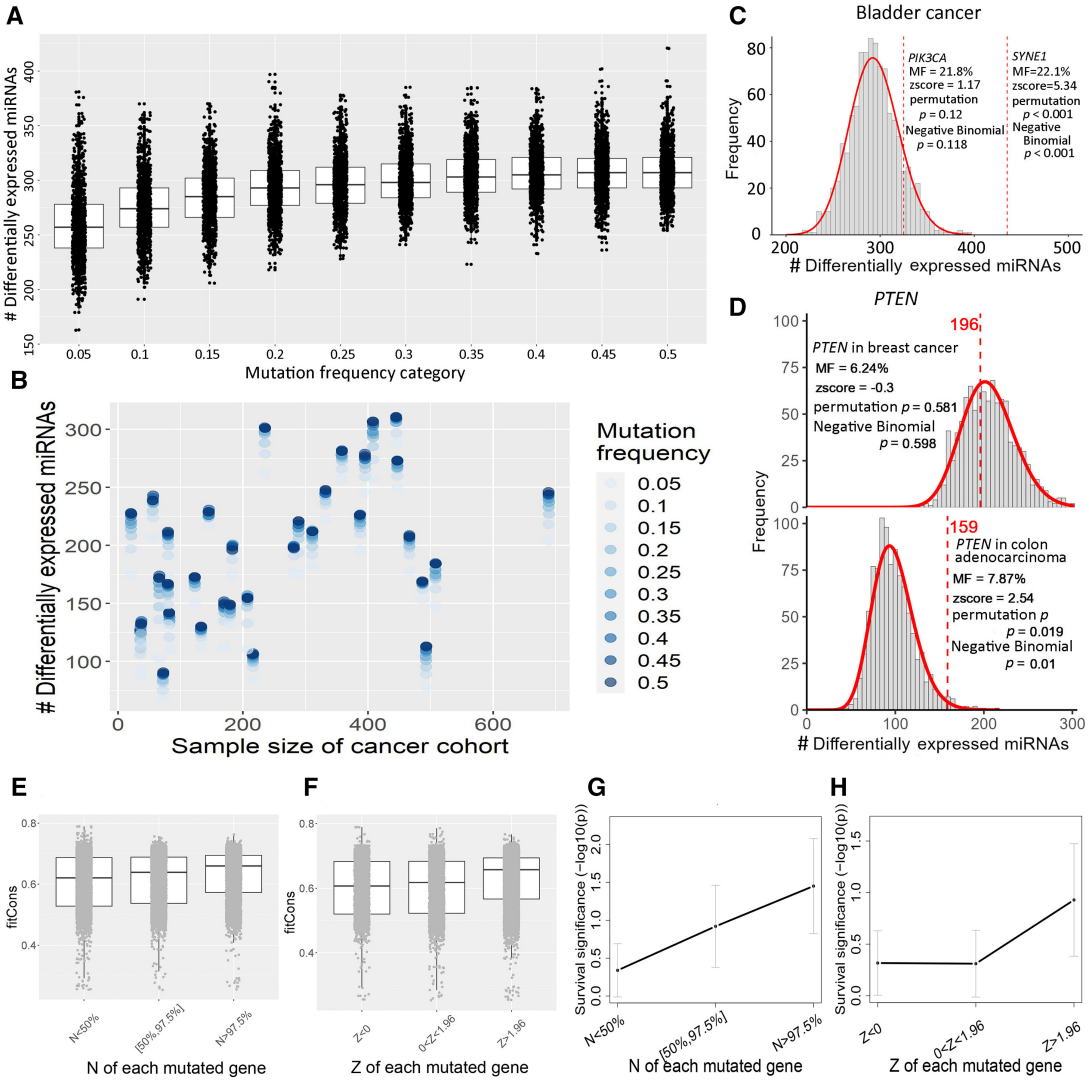
Binary mutation state labels were permutated to survey false discovery miRNAs. (A) Quantities of falsely discovered dysregulated miRNAs in the class-label-permutated bladder cancer dataset. The mutation frequencies (percentage of the positive patients) were simulated from 0.05 to 0.5 at an increment step of 0.05. Each discrete mutation frequency category contained 1000 data points. Most other cancer types showed similar trends as in bladder cancer. (B) Across 33 cancer types, the false discovery magnitude increased with bigger sample size and lesser class imbalance. (C) Two genes (*PIK3CA* and *SYNE1*) had similar mutation frequency values in bladder cancer, and thus they were subjected to one same permutation-based null distribution. (D) *PTEN'*s mutations in breast cancer and colon adenocarcinoma affected expression of 196 and 159 miRNAs, respectively. The statistical tests deemed the former an insignificant multitude whereas the latter a significant one. (E, F) Fitness consequence (fitCons) score increased with number (E) and *z*-score (F) of dysregulated miRNAs. Each dot corresponded to a mutation context (a mutated gene in a cancer type). (G, H) survival significance of gene mutation increased with number (G) and *z*-score (H) of dysregulated miRNAs. The median survival significance (−log(*P*)) at three ordinal categories by N or Z were connected in black lines. Median absolute deviations at the three categories were shown as error bars.

In a similar previous study (Ping *et al.* 2020) on the associations between mRNA expression and mutation, the number of dysregulated protein-coding genes was used as a proxy for the impact of a gene’s mutation. Such an approach ignores the potential false positives due to statistical bias as explained earlier. To demonstrate such biases, we first generated a statistical background from 1000 random permutations of the mutation state labels (mutated versus wild-type) across the patients. By conducting permutation at incremental mutation frequencies (5% up to 50%, at 5% interval) followed by DE analyses, a pattern of incremental discovery entities was clearly observable (Fig. 2A). Because of the random labeling, the statistically significant entities were considered false positives in the biological sense. The same general trend of false discoveries increasing with mutation frequency was observed for all 33 cancer types (Fig. 2B). Furthermore and intuitively, the multitude of false discoveries was also associated with sample size (Pearson correlation coefficient 0.42, *P* = 1 × 10^−15^). By comparing the actual number of DE miRNAs to the null distribution, we evaluated the unbiased associations between mRNA mutation and miRNA expression, which were unsusceptible to class imbalance and sample size. Thus, only mutation contexts that passed the statistical test criterion (*P* < .05) were considered broadly impacting miRNA expression, which was further examined for prognosis relevance based on the collective expression of their responsive miRNAs.

Our approach exerted one permutation procedure for each discrete mutation frequency level in each cancer cohort. For each mutated gene, its actual number of dysregulated miRNAs was scrutinized against the null distribution of the matching mutation frequency. For example, both *PIK3CA* and *SYNE1* were mutated in around 22% of bladder urothelial carcinoma (bladder cancer) samples. Therefore, the two responsive miRNA numbers were compared against the same null distribution constructed from permutation of bladder cancer data (1000 iterations, 20% mutant versus 80% wild-type). The DE analysis based on *PIK3CA*s mutation status yielded 325 significant miRNA. However, based on the proposed permutation procedure, a multitude of 325 mapped to a *P* = .12, suggesting that the number of DE miRNAs caused by *PIK3CA* was not sufficient to reject the hypothesis of null mutation impact (Fig. 2C). In contrast, at the same mutation frequency, *SYNE1*s mutation status produced 435 DE miRNAs, which was significant in relation to the same null distribution (Fig. 2C, *P* < .001).

From a pan-cancer perspective, a greater number of dysregulated miRNAs did not always result in a smaller *P*-value. For example, *PTEN* in colon adenocarcinoma was mutated at 7.87%. The null distribution of DE miRNAs from 1000 permutation iterations numbered 98.3 ± 23.8, accrediting *PTEN*s 159 responsive miRNAs with statistical significance (Fig. 2D, *P* = .01). In breast invasive carcinoma (breast cancer), *PTEN* has a mutation frequency similar to colon adenocarcinoma, 6.24%. Probably due to the considerably larger sample size (755 versus 409), The null distribution of DE miRNAs in breast cancer scaled to 205.1 ± 30.0, consequentially dimming *PTEN*s 196 responsive miRNAs and failing its statistical test (Fig. 2D, *P* = .60).

In summary, our permutation procedure addressed DE pitfalls associated with variant sample size and mutation frequency by effectively subjecting the ostensible responsive miRNA quantity to cancer-specific, sample-structure-controlled statistical tests.

### 3.2 Gene mutations causing more dysregulated miRNAs are associated with greater biological significance

Given the number of dysregulated miRNAs and the permutation-adjusted *P*-value for each mutation context, one can compare and rank mutated genes in the same cancer type. Such comparisons assume gene mutations that result in more dysregulated miRNAs are associated with greater biological significance. We verified this assumption in two different ways. On one hand, we employed the fitness consequence score (fitCons) (Huang *et al.* 2017) to quantitatively assess the functional impact of individual mutations, and then aggregated the fitCons score at the gene level by taking the average of all mutations located in the gene body. FitCons captures the importance of variants from a unique perspective and its measurement showed the least correlation with a panel of deleteriousness prediction scores (Liu *et al.* 2020). When the mutated genes were stratified into three groups of increasing number or *z*-score of dysregulated miRNAs, the median fitCons score per group increased (Fig. 2E and F). The fitCons scores of genes with conspicuous *z*-scores (1.96 or higher) were significantly higher than those of genes with inconspicuous *z*-scores (Wilcoxon test, *P* < 1 × 10^−15^ for group of *z* > 1.96 versus group of *z* in [0, 1.96]). At the gene level, the fitCons score showed a statistically significant correlation with both the *z*-score and the number of dysregulated miRNAs, with Pearson correlation coefficient *ρ *= 0.15 and *ρ *= 0.09, respectively (*P* < 1 × 10^−15^).

On the other hand, we assessed the survival significance of each gene’s mutation status via log-rank test. In the same manner, we stratified mutated genes to three groups by either number or *z*-score of dysregulated miRNAs. Likewise, the median survival significance increased with either number or *z*-score of dysregulated miRNAs (Fig. 2G and H). Taken together, stronger variant deleteriousness and survival significance were associated with a greater number of dysregulated miRNAs per gene. From two distinct perspectives, we verified that gene mutations causing more dysregulated miRNAs are associated with greater biological significance, thus undoubtedly advocating the permutation-adjusted statistics of miRNA dysregulation extent, including *z*-score and modified *P*-value.

### 3.3 Overall description of pan-cancer miRNA dysregulation analyses

**Figure 3.**
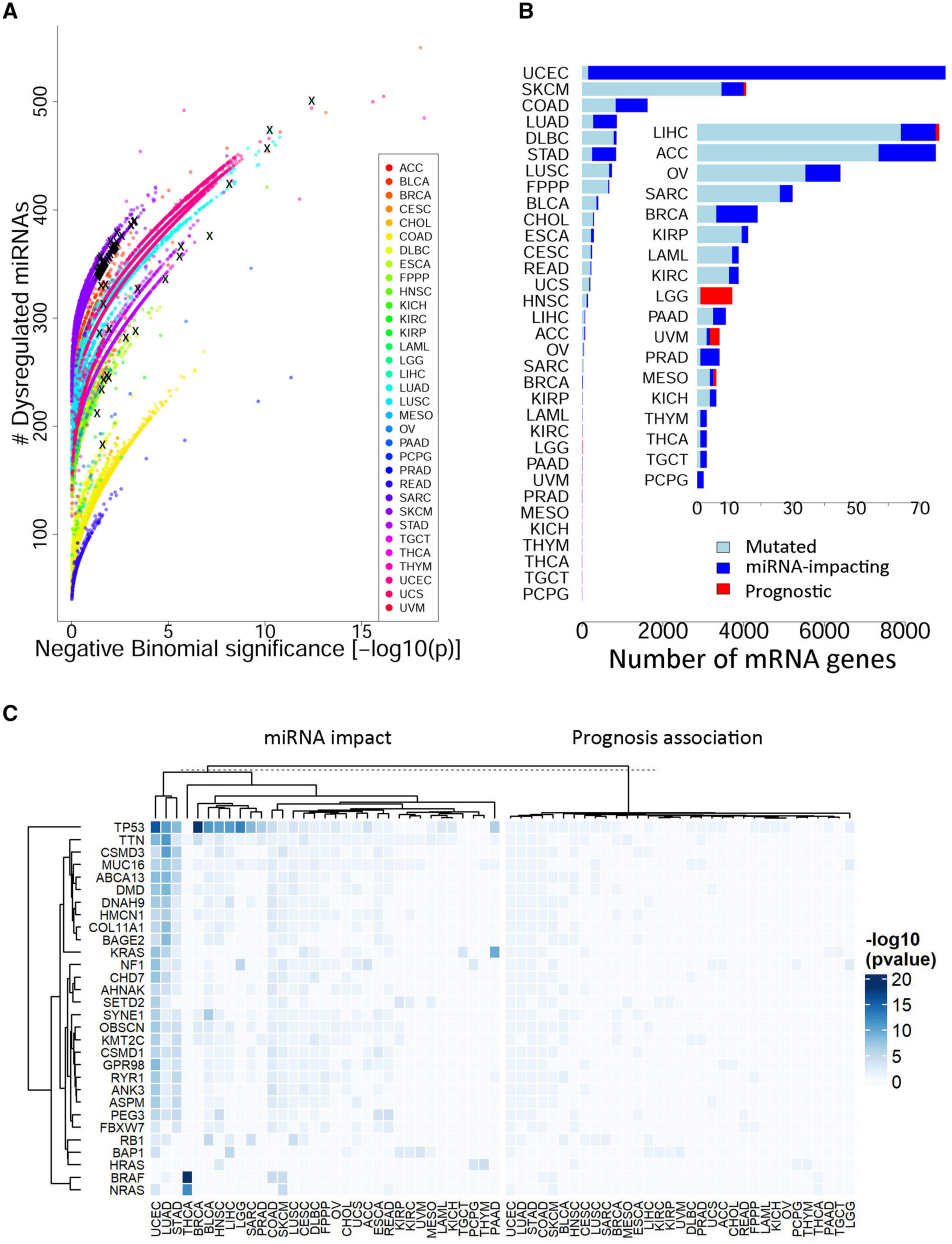
Overall description of mRNA–miRNA associations. (A) Scatterplot depicts two statistics out of DE analysis for each mutation context: number of dysregulated miRNAs and corresponding *P*-value inferred from Negative Binomial approximation of permutations. A spectrum of rainbow colors was utilized to distinguish different cancer types. Overall, the conversions from responsive miRNAs numbers to permutation-based *P*-values followed different momentums in different cancer types. Mutation contexts with overall survival significance were highlighted with black crosses. (B) Protein-coding genes (mRNAs) that were mutated in 5% or more patients underwent miRNA DE analyses, and mutated mRNA genes with abundant responsive miRNAs (a.k.a. miRNA-impacting genes, *P* < .05) further underwent aggregative prognostic analysis. The 18 cancer types of lowest quantities were magnified in the inlet plot. Of each light-blue bar representing all *mutated* genes, *miRNA-impacting* genes (former analysis) and *prognostic* genes (latter analysis) were denoted in dark blue and red, respectively. By definition, the former set is a superset of the latter set, meaning that a dark blue segment actually encompasses the corresponding red segment. (C) Thirty-three cancer types (columns) were characterized by miRNA dysregulation and prognosis association of 30 mRNA genes (rows). The mRNA genes were selected as those dysregulating miRNA expression in a great number of cancer types (*n* = 24) or in the few dissimilar cancer types (*n* = 6): READ (represented by *FBXW* and *PEG3*), THCA (*NRAS* and *BRAF*), THYM (*HRAS*), and UVM (*BAP1*). For full names of the 33 cancer types, please refer to Supplementary Text S1.

Of the 20 804 mutation contexts that returned significant DE miRNAs (DE, *P* < .05), 11 928 cases passed the permutation-based statistical tests of the miRNA dysregulation impact (modified permutation, *P* < .05), involving 9006 mRNA genes in 33 cancer types (Fig. 3A and B). Uterine corpus endometrial carcinoma (uterine cancer), colon adenocarcinoma, and skin cutaneous melanoma (skin melanoma) harbored the most miRNA-dysregulating genes, with total numbers 8849, 786, and 607, respectively. The great number of miRNA-impacting genes in uterine cancer is consistent with its hypermutation nature (Jiang *et al.* 2022).

The further prognostic analyses of the collective miRNA expression refined the miRNA-impacting mutation contexts to prognosis-associated scenarios (Beta distribution, *P* < .05, Fig. 3A and B), which numbered 83 for OS and 215 for DSS, respectively. For the OS analysis, the 83 significant mutation contexts involved 83 distinct genes in nine cancer types: bladder cancer, esophageal carcinoma, head and neck squamous cell carcinoma, brain lower grade glioma (abbreviated as LGG hereafter), liver hepatocellular carcinoma, lung squamous cell carcinoma, mesothelioma, skin melanoma, and uveal melanoma. For the DSS analysis, the 215 significant mutation contexts involved 215 distinct genes in six cancer types: bladder cancer, breast cancer, esophageal carcinoma, LGG, skin melanoma, and uveal melanoma. In terms of OS, the following three cancer types harbored the most prognosis-associated genes: skin melanoma (*n* = 61), LGG (*n* = 10), and uveal melanoma (*n* = 3); in terms of DSS, the three most prominent cancer types changed to skin melanoma (*n* = 187), bladder cancer (*n* = 13), and LGG (*n* = 9). Of note, nearly all miRNA-impacting genes in LGG, i.e. 10 (for OS) or 9 (for DSS) out of 10, were found prognostic using the CGES-summed expression of responsive miRNAs.

We identified 24 genes that dysregulated abundant miRNAs in the largest numbers of cancer types and supplemented them with six genes representative of four dissimilar cancer types missed by the former 24 genes (Fig. 3C). *TP53*, *TTN*, and *ABCA13* are ranked as the top three most pan-cancer miRNA-impacting genes, associated with 22, 9, and 8 cancer types, respectively. The 30 representative genes covered a few that showed prognosis by collective miRNA expression, including *TP53* (Beta distribution, *P* = .03), *MUC16* (*P* = .01), and *NF1* (*P* = .01) for LGG, *RYR1* (*P* = .03) for bladder cancer, *TTN* for mesothelioma (*P* = .04), and *BAP1* for uveal melanoma (*P* = .04). By manifesting broad miRNA expression impacts for nearly all these 30 representative genes, uterine cancer, lung adenocarcinoma, and stomach adenocarcinoma formed a cluster with distance from the mass cancer types (Fig. 3C).

**Figure 4.**
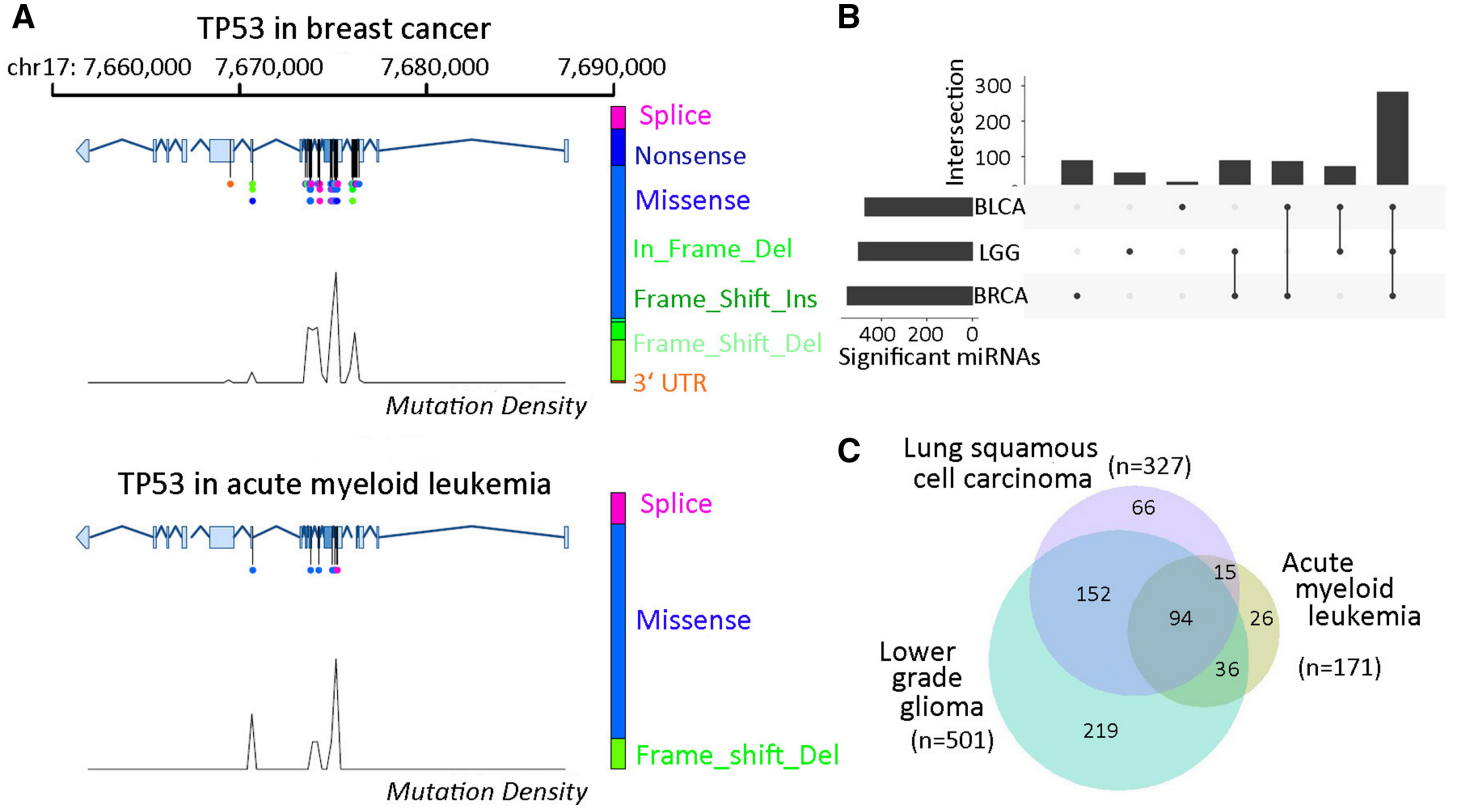
Cross-cancer comparison analyses of *TP53'*s mutations and responsive miRNAs. (A) Representative gene-mutation Ideograms for *TP53* in two cancers: *TP53'*s mutations happened at more loci and led to a greater variety of protein-coding disruptions in breast cancer as compared to acute myeloid leukemia. In one Ideogram, the gene body is illustrated in a horizontal model showing transcription start/end sites and exons. Point mutations are indicated as lollipops with various colors for different mutation types. Proportions of all mutation types and their color mapping are indicated by the vertical bar on the right. (B) A representative UpSet intersection plot: *TP53'*s mutations dysregulated the most miRNAs in breast cancer (BRCA), lower grade glioma (LGG), and bladder cancer (BLCA). A majority of miRNAs were commonly responsive across the three cancer types. (C) A proportional Venn diagram: a sizeable portion of miRNAs were commonly responsive to *TP53* mutations in three cancer types of disparate responsive miRNA multitudes.

We use the most conspicuous gene *TP53* to demonstrate some analytic graphics offered in the online portal MutMix. Gene-mutation Ideograms deploy the same gene-body frame for multiple cancer types, where the different dispersals of cancer-specific mutations are revealed (Fig. 4A). Intersection visualization stresses the multitude of shared responsive miRNAs (*n* = 283) across *TP53*'s three most influenced cancer types (Fig. 4B): breast cancer (*n* = 550), LGG (*n* = 501), and bladder cancer (*n* = 500). If we consider LGG, lung squamous cell carcinoma, and acute myeloid leukemia, i.e. three cancer types of disparate scales of miRNA totals for *TP53*, the commonly responsive miRNAs still occupy substantial portions, most notably accounting for 55% of total 171 *TP53*-responsive miRNAs in acute myeloid leukemia (Fig. 4C).

### 3.4 Mutation impacts on miRNA expression in brain lower grade glioma

Although pan-cancer analysis results were generated and documented in MutMix, due to the limitation of space, we herein focus on significant findings related to glioma. Because of the lack of miRNA data for glioblastoma, the present study covers LGG only. In total, 11 mutated genes were eligible for miRNAs DE analysis in LGG. Remarkably, all mutated genes except *TTN* were ascertained as broadly impacting miRNA expression upon mutation (permutation, *P* < .05; Table 1). *TTN* is known for its long gene size for harboring abundant mutations, thus is frequently admixed in mutation analysis results. Nine out of the 10 miRNA-impacting regulators, namely *ATRX, CIC, EGFR, FUBP1, IDH1, NF1, NOTCH1, PIK3CA*, and *TP53*, were incorporated into a conclusive molecular signature to stratify LGG into three prognosis subtypes (Cancer Genome Atlas Research Network *et al.* 2015). Compared to previous works, the current work unprecedently revealed the broad impacts of these genes on miRNA transcriptomes. While some studies have underscored the somatic mutations of the few mRNA genes and other studies have pinpointed the prognostic value of individual miRNAs, we for the first time investigated and statistically affirmed broad miRNA impacts of ten crucial mRNA genes in LGG. Comparing the different cancer types, one can see that LGG has the highest proportion of miRNA-impacting genes in mutated genes (10 out of 11) and the highest proportion of aggregately OS-prognostic genes in miRNA-impacting genes (10 out of 10). In contrast, generally, a much lower proportion of mutated genes exerted broad miRNA dysregulation effects in other cancer types. For example, 4063 genes were mutated in skin melanoma, but only 607 (14.9%) were found broadly impacting miRNA transcriptomes, among which 61 (10.0%) were aggregately prognostic for OS (Fig. 3B).

**Table 1.** Ten of 11 mutated genes broadly dysregulated miRNA transcriptomes in lower grade glioma.

Gene	Mutation frequency	No. miRNAs	miRNA dysregulation			Aggregative prognosis			
			*Z*-score	Permutation *P*	Hazard ratio		Cox *P*	CGES *P*	Beta *P*
*TP53*	48%	501	9.8	3.9E-13	3.4 [2.4–4.9]		9.8E−12	.01	.03
*IDH1*	77%	474	8.7	5.7E-11	3.9 [2.7–5.6]		7.3E−14	.001	.01
*ATRX*	38%	457	8.4	7.8E-11	3.5 [2.4–5.0]		5.9E−12	.004	.02
*CIC*	22%	424	7.4	7.0E-09	3.9 [2.7–5.6]		1.4E−13	.001	.02
*FUBP1*	9%	366	5.8	2.2E-06	3.4 [2.3–4.8]		1.3E−11	.003	.03
*EGFR*	7%	357	5.9	2.6E-06	3.7 [2.6–5.4]		4.2E−13	.001	.01
*NF1*	6%	336	5.3	1.4E-05	3.6 [2.5–5.2]		9.4E−13	.001	.01
*NOTCH1*	7%	288	3.9	5.0E-04	3.6 [2.5–5.1]		3.3E−12	.001	.01
*PIK3CA*	9%	247	2.3	1.5E-02	3.2 [2.2–4.6]		7.1E−11	.002	.03
*MUC16*	7%	212	1.8	4.8E-02	3.3 [2.3–4.8]		4.8E−11	.005	.02
*TTN*	14%	166	−0.2	5.6E-01	NA		NA	NA	NA

**Figure 5.**
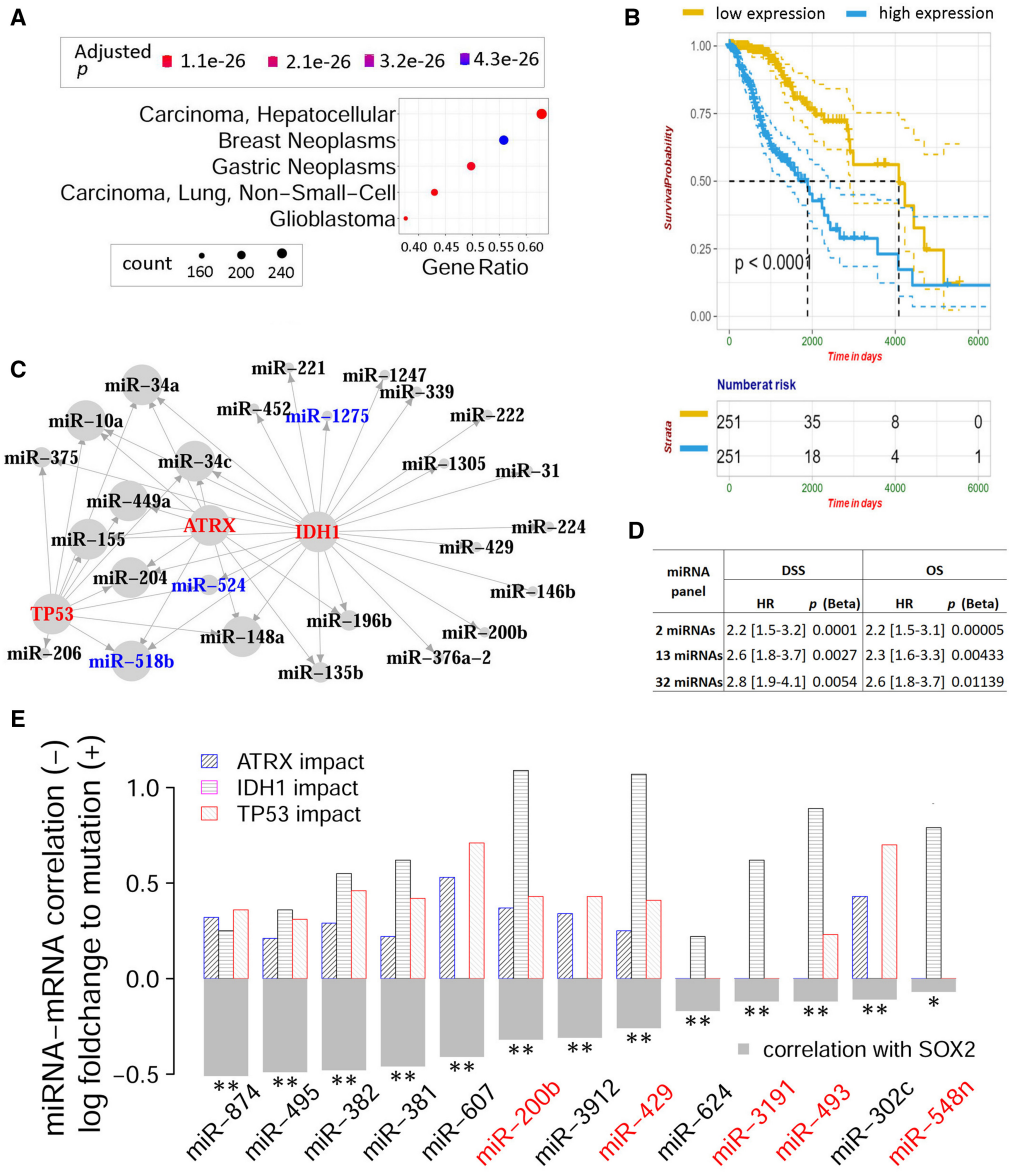
*IDH1* mutation caused prognostic miRNA responsive expression in lower grade glioma. (A) Glioblastoma was ranked the second most enriched disease of *IDH1*s responsive miRNAs in terms of *P*-value (see shade legend on top). Gene ratio equated to ratio of miRNAs known to be associated with a disease. (B) Collective expression level of *IDH1*s responsive miRNAs was predictive of overall survival, with Cox *P*-value lower than 1 × 10^−4^. (C) Network visualization of the extreme miRNA dysregulations responsive to mutations in three genes (red vertices): *TP53, IDH1*, and *ATRX*. Shown miRNAs were all related to glioblastoma and the directed edges indicated absolute log-fold changes of 1 or above. Mutations caused almost exclusively up-regulations, except to three miRNAs which were highlighted in blue vertices. Vertices were shown in three discrete sizes in accordance with the in-coming degree. (D) Aggregative prognosis analysis results using three subsets of SOX2-targeting miRNAs. The superset of 32 miRNAs was responsive to mutations of *TP53, IDH1*, or *ATRX* and were putative regulators of SOX2. The interim set of 13 miRNAs was enumerated in E, and the subset of 2 miRNAs included miR-200b and miR-429. (E) Thirteen miRNAs were significantly upregulated at mutations of the three master regulators: *TP53*, *IDH1*, and *ATRX*, and they displayed exclusive anticorrelations with *SOX2* mRNA levels. MiRNAs highlighted in red conferred individual prognostic significance. Asterisks denoted statistical significance of Pearson coefficient anticorrelation: **P < .01 and *P < .1.

*TP53, IDH1*, and *ATRX* ranked first, second, and third in terms of both the number of dysregulated miRNAs (501, 474, and 457) and the companion permutation test results (permutation, *P* = 3 × 10^−13^, 5 × 10^−11^, and 7 × 10^−11^). *TP53* is a transcription factor and a famous pan-cancer tumor suppressor gene, mutant *IDH1* drives metabolic reprogramming, redox imbalance, and causes genome-wide epigenetic alterations, including impairment of histone demethylation which results in a block to cell differentiation in glioma (Han *et al.* 2020), and *ATRX* is a regulator of chromatin remodeling and transcription (Nandakumar *et al.* 2017). Here, from the perspective of dysregulated miRNA transcriptome, the crucial roles of these regulator genes in LGG tumorigenesis and progression were corroborated. The following result descriptions were somewhat focused on *IDH1*, but the complete sets of results for *TP53* and *ATRX* can be extracted from MutMix. First, 412 of the total *IDH1*-responsive miRNAs were linked to known human diseases, and glioblastoma was ranked as the second most relevant disease, claiming 155 disease-related miRNAs (hypergeometric, *P* = 2.1 × 10^−30^, false discovery rate 3.6 × 10^−28^; Fig. 5A). Second, the *IDH1*-responsive miRNAs conferred aggregative prognostic value for LGG, with CGES permutation *P* = .001 and Beta-adjusted *P* = .01 (Fig. 5B). Third, when we restricted the known glioblastoma-related miRNAs to the extremely responsive ones (above 2-fold changes), we found *ATRX*-responsive or *TP53*-responsive miRNAs were most likely responsive to *IDH1* as well (Fig. 5C). Finally, we studied the mutation impacts of *TP53/IDH1/ATRX* on *SOX2*-targeting miRNAs. *SOX2* encodes a member of the SRY-related HMG-box (SOX) family of transcription factors, which is required for stem-cell maintenance in the central nervous system. Overall, 32 miRNAs responsive to *TP53, IDH1*, or *ATRX* mutations were considered putative regulators of *SOX2* by miRDB (Wong and Wang 2015), and they were narrowed down to 14 miRNAs that were moderately yet significantly up-regulated at mutations of one of the three regulator genes. Strikingly, 13 out of these 14 miRNAs showed anticorrelation with *SOX2*s mRNA levels in LGG patients (Fig. 5E). These 13 miRNAs were subjected to synergistic regulations from *TP53, IDH1*, and *ATRX*, showing consistent up-regulation changes upon their mutations. Compared to *TP53* and *ATRX, IDH1* seemed more influential on the 13 miRNAs, considering both width and intensity of the regulations. Of note, the most intensive up-regulations occurred on miR-200b and miR-429 in response to mutations of *IDH1*. We conducted aggregative CGES survival analyses at three successive levels, recruiting 32, 13, and 2 miRNAs, respectively (Fig. 5D). At each level, these *SOX2*-targeting and *TP53/IDH1/ATRX*-susceptible miRNAs were collectively predictive of patient OS and DSS survival (CGES permutation, *P* < .05); interestingly, the statistical significance increased as we narrowed down to fewer yet more relevant miRNAs: *P* = .011 (OS) and .005 (DSS) for 32 miRNAs, *P* = .0043 (OS) and .0027 (DSS) for 13 miRNAs, and *P* = .00005 (OS) and .0001 (DSS) for only 2 miRNAs (miR-200b and miR-429).

**Figure 6.**
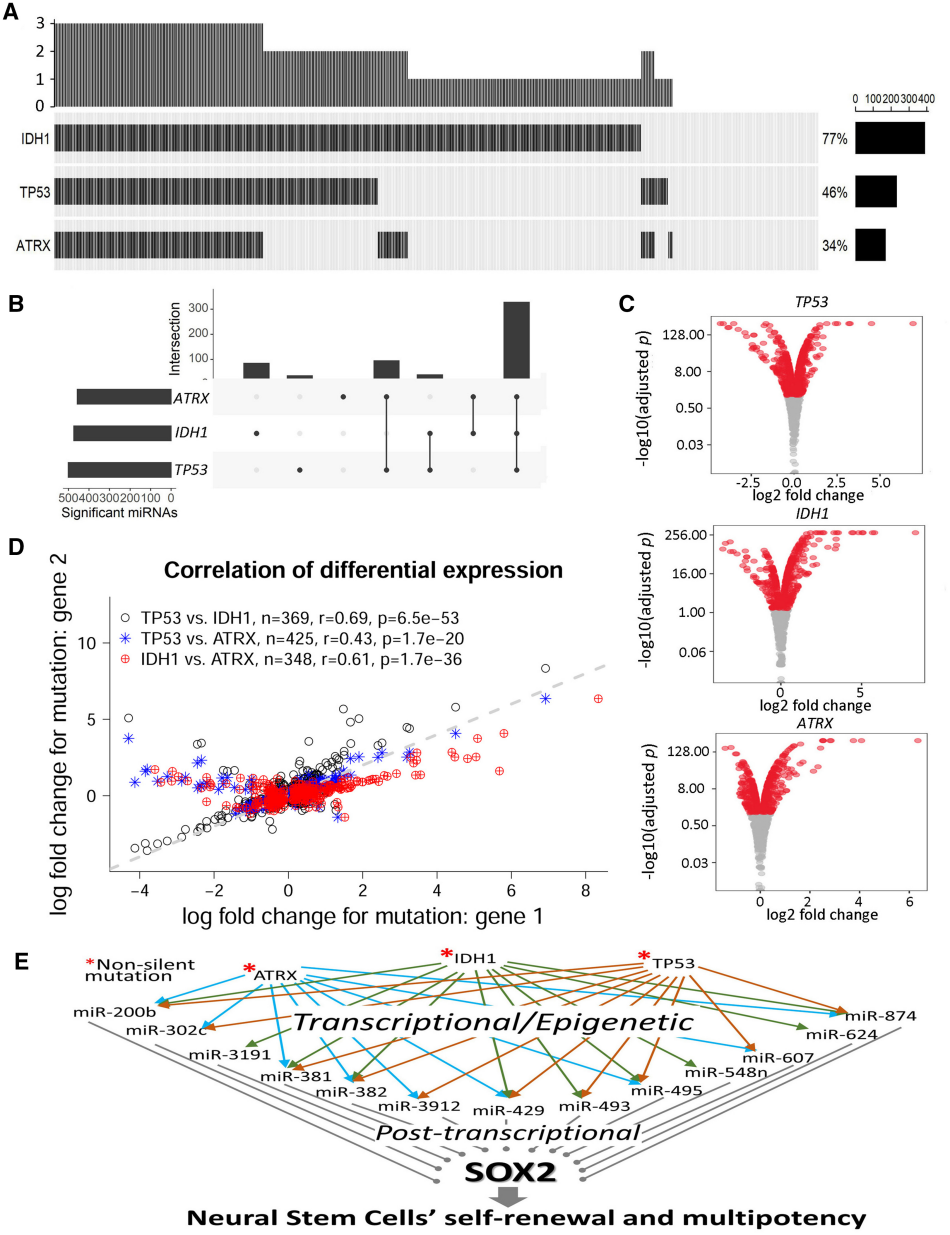
Three master regulators of miRNA expression in LGG: *TP53*, *IDH1*, and *ATRX*. (A) Oncoprint showed landscape of mutations of three genes in the cohort of 508 LGG patients. In the OncoPrint, the whole horizontal spectrum designate all patients of a cancer cohort, and a black barline indicates a mutation observed in a patient for a specific gene (row label). The mutation number (people carrying mutations for any considered gene) is represented in an overall barplot on the top. The proportion of patients carrying mutation for a particular gene is reflected in the barplot on the right. (B) The three master regulators shared a large portion of commonly responsive miRNAs. (C) Volcano plots of miRNA DE analysis results under binary divisions of patients based on mutation states of *TP53*, *IDH1*, and *ATRX*, respectively. (D) Scatterplot of miRNA expression changes for one gene’s mutation versus another gene’s mutation. Three comparisons formed among three mutation genes were overlaid in the same plot with distinct colors and shapes. The dashed gray line designated the diagonal line of *y* = *x*. “Gene 1” designates the gene specified before “versus,” and “gene 2” designates the one after “versus.” For example, in the phrase “TP53 versus IDH1,” *TP53* is gene 1, and *IDH1* is gene 2. (E) In the postulated mechanism of coordinate transcriptional/epigenetic regulations and posttranscriptional regulations, mutation effects of upstream genes *TP53*, *IDH1*, and *ATRX* in glioma patients were diffused over 13 upregulated miRNAs and thereby achieved repression of *SOX2*, the master regulator of neural stem cells’ self-renewal and multipotency.

In terms of mutation frequency (Fig. 6A) and miRNA impact (Fig. 6B), *IDH1, TP53*, and *ATRX* are the three most conspicuous regulator genes in LGG. As the most common gene mutation found in cancer cells, *TP53* dictates many cancer types and is a driver in LGG and glioblastoma (Brennan *et al.* 2013, Cancer Genome Atlas Research Network *et al.* 2015, Ceccarelli *et al.* 2016). For more than a decade, *IDH* mutations have been consistently appreciated as a critical prognosis marker in LGG (Yan *et al.* 2009, Houillier *et al.* 2010, Cancer Genome Atlas Research Network *et al.* 2015), and *ATRX* followed it to become another potential therapeutic target (Liu *et al.* 2012, Cancer Genome Atlas Research Network *et al.* 2015, Haase *et al.* 2018). While *IDH1* is most frequently mutated in LGG patients, concurrent *TP53* and *ATRX* mutations were also commonly seen in glioblastomas. A synergistic mechanism among *IDH1, TP53*, and *ATRX* was shown to block the differentiation of human neural stem cells via transcriptional repression of SOX2 (Modrek *et al.* 2017). Here, our analysis of mutation-associated miRNA disruptions revealed the cooperation of the three master regulators in regulating the miRNA transcriptome. The three regulators each affected abundant miRNAs and a majority of the susceptible miRNAs were targeted by all three (Fig. 6B and C). With respect to the common susceptible miRNAs, mutations of *TP53, IDH1*, and *ATRX* generally exerted consistent miRNA up-regulation impacts (Fig. 6D). While *TP53* mutations led to a coarse balance between up-regulations and down-regulations of miRNAs, *IDH1* and *ATRX* caused more up-regulations than down-regulations of miRNAs (Fig. 6C). Compared to *TP53* and *ATRX, IDH1* exerted stronger miRNA up-regulations (Fig. 6D) and had more exclusive affectees of intensive up-regulations (Fig. 5C). These results indicated a superior status of *IDH1* over other LGG regulators, which is consistent with an independent postulation that *IDH1* mutation was an initiative event in LGG, preceding *TP53* and *ATRX* mutations (Modrek *et al.* 2017).

Overall, our results suggested that mutations of LGG regulator genes caused broad, synergistic miRNA dysregulations, thereby diffusing oncogenic effects to key players of important cellular pathways. miRNAs are important posttranscriptional regulators and they enable agile tuning of cellular transcripts of most genes. Some of our identified miRNA disruptions have been characterized with clinical significance in glioma. For example, high expression of miR-155, a downstream event of *TP53/IDH1/ATRX* mutations (Fig. 5C), has been linked to poor OS (Zhou *et al.* 2019). Moreover, as a key oncogenic miRNA in glioblastoma pathogenesis (Aloizou *et al.* 2020), miR-21 was significantly up-regulated by *IDH1* mutations (log-fold change 0.86, adjusted *P *=* *4.5 × 10^−77^). In addition, of a five-miRNA prognostic signature (Zhang *et al.* 2020), all miRNAs (miR-148a, miR-155, miR-196b, miR-10b, and miR-15b) were up-regulated by at least one of the three master regulators, and three (miR-148a, miR-155, and miR-196b) showed 2-fold or higher up-regulations (Fig. 5C). Surrounding *SOX2*, the master regulator of neural stem cell self-renewal that plays a critical role also in glioblastoma stem-like cells (Suvà *et al.* 2014, Balça-Silva *et al.* 2017, Singh *et al.* 2017), we identified 13 miRNAs that were upregulated upon *TP53/IDH1/ATRX* mutations and anticorrelated with *SOX2* transcripts (Fig. 5E). Consequentially, our results brought about a coherent pretranscriptional and posttranscriptional mechanism where around a dozen mutation-upregulated miRNAs cooperated to repress *SOX2* and further inhibited neural stem cells’ self-renewal and multipotency (Fig. 6E). This mechanistic postulation was envisioned in LGG with the most coherent transcriptomic evidence, but similar mechanisms may be postulated from data on other cancer types available in MutMix.

## 4 Discussion

In cancer research, gene mutations are critical molecular markers to facilitate diagnosis and prognosis. Furthermore, transcriptome changes correlated with gene mutations are even more strong evidence to guide research and/or clinical practices. The correlated knowledge of gene mutations and expression consequences is unevenly accumulated across various cancer types. Hence, pan-cancer, multiomics approaches to public consortium data is highly pursued to enhance our knowledge of the transcriptomic expression consequences of gene mutations. In the present study, pan-cancer, multiomics approaches were employed on TCGA data to investigate cascading effects of somatic mutation over miRNA dysregulation network. At the core of our analysis results are mRNA–miRNA associations estimated from a wide panel of cancer types separately. While we surmise the miRNA dysregulation is a downstream effect of mRNAs mutation, we admit that the identified associations between protein-coding genes (mRNAs) and miRNAs are rather statistical than causal. To highlight more plausible causal associations among the results, we included information from TransmiR and signaling pathway databases to complement the mechanistic explanation. Nevertheless, oftentimes it is hard to convincingly establish causative connections between somatic mutations in regulatory genes and target expression changes, and this remains as a limitation of the present study awaiting future improvement.

LGG was the most remarkable cancer type because 10 of its 11 mutated genes all broadly perturbed miRNA transcriptomes. In LGG, details on expression responses, miRNA cotargeting, and prognosis relevance were expounded for three master regulators: *TP53*, *IDH1*, and *ATRX*. Previous work has stressed that these three genes tend to be comutated in LGG with prognostic significance (Jiang *et al.* 2022), and their cooperative mechanism was elucidated at epigenetic/transcriptional level towards SOX2. Nevertheless, contrary to our expectation, we found the LGG patients’ genomic and clinical data of TCGA did not certify SOX2 as a prognostic marker at the transcript level. Because our data revealed *TP53/IDH1*/*ATRX* joint targeting on the miRNA transcriptome and the aggregative prognostic value via the susceptible miRNAs, we came up with a posttranscriptional mechanism where mutated *TP53*, *IDH1*, and *ATRX* cooperate to inhibit SOX2 via a core set of 13 SOX2-targeting miRNAs. If *SOX2* is indeed subjected to such a posttranscriptional mechanism, its transcript level may not directly reflect its functional activity, which explains the lack of prognosis significance in *SOX2* expression level. This is a concrete example of somatic mutation effects being diffused over miRNA dysregulations. These results may guide in-depth studies of epistatic effects among the three master regulators and possibly translate the research results to diagnostic, prognostic, or therapeutic directions.

In the end, we developed MutMix as the first online portal on mutation-associated miRNA dysregulations and their relevance with disease and survival phenotypes. We organized the enormous mRNA–miRNA association data generated from our improved statistical framework in four sectors to allow for exploration and maneuvering in an online portal. In the Mutation dimension, MutMix renders oncology graphics (OncoPrints) and gene-mutation Ideograms to dissect cancer mutations from distinct perspectives. In the Association dimension, the extent of miRNA dysregulation is statistically assessed through permutation for each mutation-borne gene, and the prognosis relevance is discerned both at the individual miRNA level and at the convergent gene level. In the Network dimension, cancer-specific pleiotropic and confluent mRNA–miRNA networks are conceptualized, allowing for both forward queries by mRNA and reverse queries by miRNA. Lastly, the Disease dimension prioritizes the most relevant human diseases that are enriched in the mutation-dysregulated miRNAs. Remarkably, MutMix is augmented with opulent annotations such as somatic motifs and evidenced mRNA–miRNA regulatory relationships. Furthermore, cross-gene/cross-cancer intersections of mRNA–miRNA associations permit contrastive analyses of miRNA dysregulations between related genes or between related cancer types. In summary, MutMix provides multiple dimensions to dissect enormous mutation-expression relations between mRNAs and miRNAs, helping to elucidate cancer mutations’ functional impact and phenotype association from a unique miRNA dysregulation perspective.

Using an improved statistical framework than previous approaches, we ascertained more than 9000 genes with impacts on abundant miRNAs in 33 cancer types. Of these numerous mutation contexts, hundreds of genes cast their mutation influence on prognosis through collective miRNA dysregulations. *TP53* was the most remarkable gene by showing broad miRNA dysregulations in 22 cancer types. Specifically, we elaborated a concrete example of *TP53/IDH1/ATRX* mutation effects being diffused over miRNA dysregulations in lower grade glioma. Our improved statistical framework and pan-cancer miRNA dysregulation results may cultivate meaningful posttranscriptional mechanistic hypotheses in a wide array of cancer types.

## Supplementary Material

btad520_Supplementary_Data

## References

[btad520-B1] Aloizou A-M , Pateraki G, Siokas V et al The role of miRNA-21 in gliomas: hope for a novel therapeutic intervention? Toxicol Rep 2020;7:1514–30. 33251119 10.1016/j.toxrep.2020.11.001 PMC7677650

[btad520-B2] Balça-Silva J , Matias D, Dubois LG et al The expression of connexins and SOX2 reflects the plasticity of glioma stem-like cells. Transl Oncol 2017;10:555–69. 28654819 10.1016/j.tranon.2017.04.005 PMC5487246

[btad520-B3] Bhattacharya A , Cui Y. SomamiR 2.0: a database of cancer somatic mutations altering microRNA-ceRNA interactions. Nucleic Acids Res 2016;44:D1005–10. 26578591 10.1093/nar/gkv1220 PMC4702864

[btad520-B4] Brennan CW , Verhaak RGW, McKenna A et al; TCGA Research Network. The somatic genomic landscape of glioblastoma. Cell 2013;155:462–77. 24120142 10.1016/j.cell.2013.09.034 PMC3910500

[btad520-B5] Cancer Genome Atlas Research Network et al Comprehensive, integrative genomic analysis of diffuse lower-grade gliomas. N Engl J Med 2015;372:2481–98. 26061751 10.1056/NEJMoa1402121 PMC4530011

[btad520-B6] Ceccarelli M , Barthel FP, Malta TM et al; TCGA Research Network. Molecular profiling reveals biologically discrete subsets and pathways of progression in diffuse glioma. Cell 2016;164:550–63. 26824661 10.1016/j.cell.2015.12.028 PMC4754110

[btad520-B7] Cerami E , Gao J, Dogrusoz U et al The cBio cancer genomics portal: an open platform for exploring multidimensional cancer genomics data. Cancer Discov 2012;2:401–4. 22588877 10.1158/2159-8290.CD-12-0095 PMC3956037

[btad520-B8] Conway JR , Lex A, Gehlenborg N. UpSetR: an R package for the visualization of intersecting sets and their properties. Bioinformatics 2017;33:2938–40. 28645171 10.1093/bioinformatics/btx364 PMC5870712

[btad520-B9] Ellrott K , Bailey MH, Saksena G et al; Cancer Genome Atlas Research Network. Scalable open science approach for mutation calling of tumor exomes using multiple genomic pipelines. Cell Syst 2018;6:271–81.e7. 29596782 10.1016/j.cels.2018.03.002 PMC6075717

[btad520-B10] Gamazon ER , Wheeler HE, Shah KP et al; GTEx Consortium. A gene-based association method for mapping traits using reference transcriptome data. Nat Genet 2015;47:1091–8. 26258848 10.1038/ng.3367 PMC4552594

[btad520-B11] Gillespie M , Jassal B, Stephan R et al The reactome pathway knowledgebase 2022. Nucleic Acids Res 2022;50:D687–92. 34788843 10.1093/nar/gkab1028 PMC8689983

[btad520-B12] Haase S , Garcia-Fabiani MB, Carney S et al Mutant ATRX: uncovering a new therapeutic target for glioma. Expert Opin Ther Targets 2018;22:599–613. 29889582 10.1080/14728222.2018.1487953 PMC6044414

[btad520-B13] Han S , Liu Y, Cai SJ et al IDH mutation in glioma: molecular mechanisms and potential therapeutic targets. Br J Cancer 2020;122:1580–9. 32291392 10.1038/s41416-020-0814-x PMC7250901

[btad520-B14] Hentze MW , Castello A, Schwarzl T et al A brave new world of RNA-binding proteins. Nat Rev Mol Cell Biol 2018;19:327–41. 29339797 10.1038/nrm.2017.130

[btad520-B15] Houillier C , Wang X, Kaloshi G et al IDH1 or IDH2 mutations predict longer survival and response to temozolomide in low-grade gliomas. Neurology 2010;75:1560–6. 20975057 10.1212/WNL.0b013e3181f96282

[btad520-B16] Huang YF , Gulko B, Siepel A. Fast, scalable prediction of deleterious noncoding variants from functional and population genomic data. Nat Genet 2017;49:618–24. 28288115 10.1038/ng.3810 PMC5395419

[btad520-B17] Huang Z , Shi J, Gao Y et al HMDD v3.0: a database for experimentally supported human microRNA-disease associations. Nucleic Acids Res 2019;47:D1013–7. 30364956 10.1093/nar/gky1010 PMC6323994

[btad520-B18] Jia P , Zhao Z. Impacts of somatic mutations on gene expression: an association perspective. Brief Bioinform 2017;18:413–25. 27127206 10.1093/bib/bbw037 PMC5862283

[btad520-B19] Jiang L , Duan M, Guo F et al SMDB: pivotal somatic sequence alterations reprogramming regulatory cascades. NAR Cancer 2020;2:zcaa030. 33094288 10.1093/narcan/zcaa030 PMC7556404

[btad520-B20] Jiang L , Guo F, Tang J et al SBSA: an online service for somatic binding sequence annotation. Nucleic Acids Res 2022;50:e4. 34606615 10.1093/nar/gkab877 PMC8500130

[btad520-B21] Jiang L , Yu H, Ness S et al Comprehensive analysis of co-mutations identifies cooperating mechanisms of tumorigenesis. Cancers (Basel) 2022;14:415. 35053577 10.3390/cancers14020415 PMC8774165

[btad520-B22] Jiang Q , Wang Y, Hao Y et al miR2Disease: a manually curated database for microRNA deregulation in human disease. Nucleic Acids Res 2009;37:D98–104. 18927107 10.1093/nar/gkn714 PMC2686559

[btad520-B23] Kanehisa M , Goto S. KEGG: Kyoto Encyclopedia of Genes and Genomes. Nucleic Acids Res 2000;28:27–30. 10592173 10.1093/nar/28.1.27 PMC102409

[btad520-B24] Liu J , Lichtenberg T, Hoadley KA et al; Cancer Genome Atlas Research Network. An integrated TCGA pan-cancer clinical data resource to drive high-quality survival outcome analytics. Cell 2018;173:400–16.e11. 29625055 10.1016/j.cell.2018.02.052 PMC6066282

[btad520-B25] Liu X , Li C, Mou C et al dbNSFP v4: a comprehensive database of transcript-specific functional predictions and annotations for human nonsynonymous and splice-site SNVs. Genome Med 2020;12:103. 33261662 10.1186/s13073-020-00803-9 PMC7709417

[btad520-B26] Liu X-Y , Gerges N, Korshunov A et al Frequent ATRX mutations and loss of expression in adult diffuse astrocytic tumors carrying IDH1/IDH2 and TP53 mutations. Acta Neuropathol 2012;124:615–25. 22886134 10.1007/s00401-012-1031-3

[btad520-B27] McGeary SE , Lin KS, Shi CY et al The biochemical basis of microRNA targeting efficacy. Science 2019;366:eaav1741. 31806698 10.1126/science.aav1741 PMC7051167

[btad520-B28] Modrek AS , Golub D, Khan T et al Low-grade astrocytoma mutations in IDH1, P53, and ATRX cooperate to block differentiation of human neural stem cells via repression of SOX2. Cell Rep 2017;21:1267–80. 29091765 10.1016/j.celrep.2017.10.009 PMC5687844

[btad520-B29] Nandakumar P , Mansouri A, Das S. The role of ATRX in glioma biology. Front Oncol 2017;7:236. 29034211 10.3389/fonc.2017.00236 PMC5626857

[btad520-B30] Ng AHM , Khoshakhlagh P, Rojo Arias JE et al A comprehensive library of human transcription factors for cell fate engineering. Nat Biotechnol 2021;39:510–9. 33257861 10.1038/s41587-020-0742-6 PMC7610615

[btad520-B31] Ping J , Oyebamiji O, Yu H et al MutEx: a multifaceted gateway for exploring integrative pan-cancer genomic data. Brief Bioinform 2020;21:1479–86. 31588509 10.1093/bib/bbz084 PMC7373173

[btad520-B32] Robinson MD , McCarthy DJ, Smyth GK. edgeR: a bioconductor package for differential expression analysis of digital gene expression data. Bioinformatics 2010;26:139–40. 19910308 10.1093/bioinformatics/btp616 PMC2796818

[btad520-B33] Samuels DC , Yu H, Guo Y. Is it time to reassess variant annotation? Trends Genet 2022;38:521–3. 35232614 10.1016/j.tig.2022.02.002

[btad520-B34] Singh DK , Kollipara RK, Vemireddy V et al Oncogenes activate an autonomous transcriptional regulatory circuit that drives glioblastoma. Cell Rep 2017;18:961–76. 28122245 10.1016/j.celrep.2016.12.064 PMC5321610

[btad520-B35] Suvà ML , Rheinbay E, Gillespie SM et al Reconstructing and reprogramming the tumor-propagating potential of glioblastoma stem-like cells. Cell 2014;157:580–94. 24726434 10.1016/j.cell.2014.02.030 PMC4004670

[btad520-B36] Tong Z , Cui Q, Wang J et al TransmiR v2.0: an updated transcription factor-microRNA regulation database. Nucleic Acids Res 2019;47:D253–8. 30371815 10.1093/nar/gky1023 PMC6323981

[btad520-B37] Tu K , Yu H, Hua Y-J et al Combinatorial network of primary and secondary microRNA-driven regulatory mechanisms. Nucleic Acids Res 2009;37:5969–80. 19671526 10.1093/nar/gkp638 PMC2764428

[btad520-B38] Wang K , Li M, Hakonarson H. ANNOVAR: functional annotation of genetic variants from high-throughput sequencing data. Nucleic Acids Res 2010;38:e164. 20601685 10.1093/nar/gkq603 PMC2938201

[btad520-B39] Wong N , Wang X. miRDB: an online resource for microRNA target prediction and functional annotations. Nucleic Acids Res 2015;43:D146–52. 25378301 10.1093/nar/gku1104 PMC4383922

[btad520-B40] Wu T , Hu E, Xu S et al clusterProfiler 4.0: a universal enrichment tool for interpreting omics data. Innovation 2021;2:100141. 34557778 10.1016/j.xinn.2021.100141 PMC8454663

[btad520-B41] Yan H , Parsons DW, Jin G et al IDH1 and IDH2 mutations in gliomas. N Engl J Med 2009;360:765–73. 19228619 10.1056/NEJMoa0808710 PMC2820383

[btad520-B42] Ye B , Shi J, Kang H et al Advancing pan-cancer gene expression survial analysis by inclusion of non-coding RNA. RNA Biol 2020;17:1666–73. 31607216 10.1080/15476286.2019.1679585 PMC7567505

[btad520-B43] Zhang J-H , Hou R, Pan Y et al A five-microRNA signature for individualized prognosis evaluation and radiotherapy guidance in patients with diffuse lower-grade glioma. J Cell Mol Med 2020;24:7504–14. 32412186 10.1111/jcmm.15377 PMC7339211

[btad520-B44] Zhou Y , Wang X, Liu Z et al Prognostic role of microRNA-155 expression in gliomas: a meta-analysis. Clin Neurol Neurosurg 2019;176:103–9. 30554090 10.1016/j.clineuro.2018.12.005

