# Supplementary material for: Somatic mutation effects diffused over microRNA dysregulation
Source: Bioinformatics. 2023 Aug 25;39(9):btad520. doi: 10.1093/bioinformatics/btad520 (PMC10474951; doi:10.1093/bioinformatics/btad520)
Supplement: btad520_Supplementary_Data [file btad520_supplementary_data.docx]

Somatic mutation effects diffused over microRNA dysregulation

Hui Yu^1,§^, Limin Jiang^1,§^, Chung-I Li^2^, Scott Ness^3^, Sara G.M. Piccirillo^3^, Yan Guo^1,*^

**Supplementary Text 1**

## **Abbreviations used in the manuscript (including acronyms for 33 cancer cohorts)**

**ACC**: Adrenocortical carcinoma; **BLCA**: Bladder Urothelial Carcinoma; **BRCA**: Breast invasive carcinoma; **CESC**: Cervical squamous cell carcinoma and endocervical adenocarcinoma; **CHOL**: Cholangiocarcinoma; **LCML**: Chronic Myelogenous Leukemia; **COAD**: Colon adenocarcinoma; **CNTL**: Controls; **ESCA**: Esophageal carcinoma; **FPPP**: FFPE Pilot Phase II; **GBM**: Glioblastoma multiforme; **HNSC**: Head and Neck squamous cell carcinoma; **KICH**: Kidney Chromophobe; **KIRC**: Kidney renal clear cell carcinoma; **KIRP**: Kidney renal papillary cell carcinoma; **LAML**: Acute Myeloid Leukemia; LGG: Brain Lower Grade Glioma; **LIHC**: Liver hepatocellular carcinoma; **LUAD**: Lung adenocarcinoma; **LUSC**: Lung squamous cell carcinoma; **DLBC**: Lymphoid Neoplasm Diffuse Large B-cell Lymphoma; **MESO**: Mesothelioma; **MISC**: Miscellaneous; **OV**: Ovarian serous cystadenocarcinoma; **PAAD**: Pancreatic adenocarcinoma; **PCPG**: Pheochromocytoma and Paraganglioma; **PRAD**: Prostate adenocarcinoma; **READ**: Rectum adenocarcinoma; **SARC**: Sarcoma; **SKCM**: Skin Cutaneous Melanoma; **STAD**: Stomach adenocarcinoma; **TGCT**: Testicular Germ Cell Tumors; **THYM**: Thymoma; **THCA**: Thyroid carcinoma; **UCS**: Uterine Carcinosarcoma; **UCEC**: Uterine Corpus Endometrial Carcinoma; **UVM**: Uveal Melanoma; **CGES**: Composite Gene Expression Score;

**DE**: Differential Expression; **DSS**: Disease-Specific Survival; **OS**: Overall Survival; **TCGA**: The Cancer Genome Atlas.
